# Methyl α-l-sorboside monohydrate

**DOI:** 10.1107/S2414314621013250

**Published:** 2021-12-21

**Authors:** Mao Matsumoto, Natsumi Nagayama, Ryo Hirose, Kei Takeshita, Tomohiko Ishii

**Affiliations:** a Department of Advanced Materials Science, Graduate School of Engineering, Kagawa University, 2217-20 Hayashi-cho, Takamatsu, Kagawa 761-0396, Japan; b Fushimi Pharmaceutical Co Ltd, 307 Minatomachi, Marugame, Kagawa 763-8605, Japan; Howard University, USA

**Keywords:** crystal structure, hydrogen bonding, rare sugar, alkyl sorboside

## Abstract

Methyl l-sorboside monohydrate, C_7_H_14_O_6_·H_2_O, was prepared from the rare sugar l-sorbose, C_6_H_12_O_6_, and crystallized. It was confirmed that methyl l-sorboside formed α-pyran­ose with a ^2^ *C*_5_ conformation and crystallized with one water molecule of crystallization. In the crystal, mol­ecules are linked by O—H⋯O hydrogen bonds, forming a three-dimensional network. The unit-cell volume of the title compound, methyl l-sorboside monohydrate, is 481.13 (2) Å^3^ (*Z* = 2), which is about 108.16 Å^3^ (29.0%) greater than that of half the amount of the chemical α-l-sorbose [745.94 (2) Å^3^ (*Z* = 4)].

## Abstract 2

Singl crystals of the title compound were synthesized by the dehydrative condensation of α-l-sorbose and methanol.

## Graphical abstract

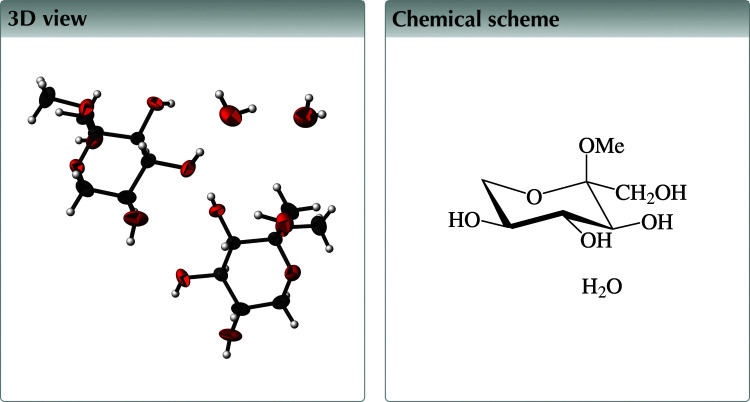

## Structure description

**Figure 1:**
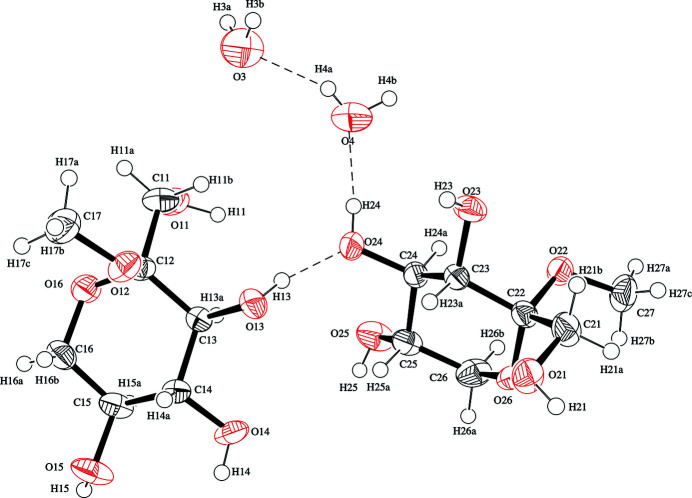
An *ORTEP* view of the title compound with the atom-labeling scheme. Displacement ellipsoids of all non-hydrogen atoms are drawn at the 50% probability level. H atoms are shown as small spheres of arbitrary radii. Hydrogen bonds are shown as dashed lines.

The rare sugar l-sorbose was the first l-form hexose found in nature (Itoh *et al.*, 1995 ▸; Khan *et al.*, 1992 ▸; Nordenson *et al.*, 1979 ▸). Methyl l-sorboside (Fig. 1 ▸) is an α-pyran­ose form in which the OH group located on the C-2 position in l-sorbose is converted into a meth­oxy group OCH_3_. The mol­ecular weight of methyl l-sorboside, C_7_H_14_O_6_·H_2_O, is 212.20. On the other hand, that of l-sorbose, C_6_H_12_O_6_, is 180. The increase in mol­ecular weight from sorbose to sorboside is thus about 18%. In this study, we aimed to produce a single crystal of methyl l-sorboside that contains sorboside mol­ecules and water mol­ecules in the ratio of 1 to 1 in the unit cell. The crystal system of ethyl l-sorboside (Nagayama *et al.*, 2020 ▸), which we reported previously, is ortho­rhom­bic, while that of methyl l-sorboside is triclinic. The space group of ethyl l-sorboside is *P*2_1_2_1_2_1_ (*Z* = 4), while that of methyl l-sorboside is *P*1 (*Z* = 2). Furthermore, concerning the crystal solvent, ethyl-l-sorboside contains no solvent mol­ecules in the crystal, whereas crystals of methyl l-sorboside contain water mol­ecules as crystallization water. Thus, methyl l-sorboside is only one mol­ecule shorter in the alkyl-carbon chain length than ethyl l-sorboside, but the crystal system, space group, and crystal solvent are significantly different.

It was confirmed that methyl l-sorboside formed an α-pyran­ose with a ^2^ *C*_5_ conformation and a water mol­ecule of crystallization. Comparing these two independent methyl l-sorboside mol­ecules, we found that the positions of the carbon and oxygen atoms are roughly the same. On the other hand, the positions of the hydrogen atoms determined from the X-ray diffraction measurement results are different, resulting in different orientations of the hy­droxy groups.

**Figure 2:**
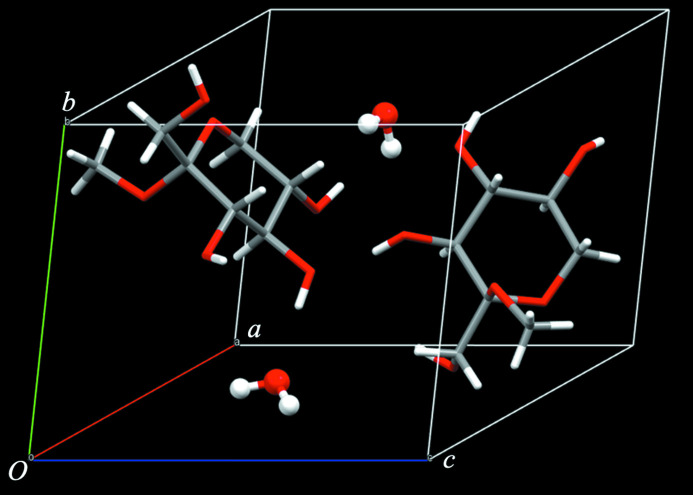
A packing diagram of the title compound. Sugar mol­ecules are shown in a framework type, whereas the crystal water mol­ecules are shown in a ball-and stick type.

Hydrogen bonds (Fig. 2 ▸, Table 1 ▸) occur between the hy­droxy groups of the methyl l-sorboside mol­ecules or through the water mol­ecules of crystallization, and the overall network extends parallel to the *ab* plane. However, the hydrogen-bond network is weak in the *c*-axis direction because the hydro­phobic meth­oxy group does not take part in any hydrogen bonds. Therefore, the three-dimensional hydrogen-bonding network has become a pseudo two-dimensional network.

## Synthesis and crystallization

Methyl l-sorboside, α-sorbo­pyran­oside form, was prepared by Fischer glycosidation from l-sorbose and methanol (Taguchi *et al.*, 2018 ▸). The Fisher method produces isomers such as α-, β-, and furan­ose. Therefore, chromatographic separation using an ion-exchange resin was performed. The reaction mixture was evaporated under vacuum at 40°C to remove the solvent and dissolved in water. Then the mixture was applied to a column of ion-exchange resins (Dowex 50W-X2, Ca^2+^ form) and was eluted with deionized water. After separation, each fraction was analysed by HPLC, and fractions containing the α-pyran­oside type were collected and concentrated to syrup. Small single crystals were obtained by placing the syrup in a Petri dish and keeping it at 4°C. It is obvious that the synthesized methyl α-l-sorboside is still in the l-form after dehydrative condensation, because l-sorbose is used as the starting material. The absolute structure wa also confirmed by the Flack parameter (Flack, 1983 ▸).

## Refinement

Crystal data, data collection and structure refinement details are summarized in Table 2 ▸.

## Supplementary Material

Crystal structure: contains datablock(s) I. DOI: 10.1107/S2414314621013250/bv4043sup1.cif

Structure factors: contains datablock(s) I. DOI: 10.1107/S2414314621013250/bv4043Isup2.hkl

CCDC reference: 2120624

Additional supporting information:  crystallographic information; 3D view; checkCIF report

## Figures and Tables

**Table 1:** Hydrogen-bond geometry (Å, °)

*D*—H⋯*A*	*D*—H	H⋯*A*	*D*⋯*A*	*D*—H⋯*A*
O4—H4*A*⋯O3	0.85	1.94	2.729 (7)	154
O11—H11⋯O21^i^	1.00 (8)	1.87 (8)	2.799 (4)	154 (6)
O13—H13⋯O24	0.82	1.83	2.643 (4)	169
O14—H14⋯O11^ii^	0.82	1.94	2.719 (4)	159
O15—H15⋯O13^iii^	0.82	2.12	2.898 (4)	158
O21—H21⋯O25^iv^	0.82	2.10	2.874 (4)	157
O23—H23⋯O14^v^	0.82	1.84	2.653 (4)	169
O24—H24⋯O4	0.82	1.83	2.650 (5)	176
O25—H25⋯O23^iii^	0.82	1.97	2.709 (5)	150

Symmetry codes: (i) 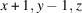 ; (ii) 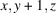 ; (iii) 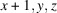 ; (iv) 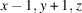 ; (v) 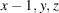 .

**Table 2:** Experimental details

Crystal data	
Chemical formula	C_7_H_14_O_6_·H_2_O
*M*_r_	212.20
Crystal system, space group	Triclinic, *P*1
Temperature (K)	296
*a*, *b*, *c* (Å)	6.7320 (5), 7.7574 (5), 10.6128 (8)
α, β, γ (°)	82.458 (6), 72.596 (5), 65.476 (5)
*V* (Å^3^)	481.13 (6)
*Z*	2
Radiation type	Cu *K*α
μ (mm^−1^)	1.15
Crystal size (mm)	0.1 × 0.1 × 0.1

Data collection	
Diffractometer	Rigaku R-AXIS RAPID
Absorption correction	Multi-scan (*ABSCOR*; Rigaku, 1995 ▸)
*T*_min_, *T*_max_	0.698, 1.000
No. of measured, independent and observed [*I* > 2σ(*I*)] reflections	5541, 2880, 2751
*R*_int_	0.045
(sin θ/λ)_max_ (Å^−1^)	0.602

Refinement	
*R*[*F* ^2^ > 2σ(*F* ^2^)], *wR*(*F* ^2^), *S*	0.048, 0.125, 1.13
No. of reflections	2880
No. of parameters	272
No. of restraints	3
H-atom treatment	H atoms treated by a mixture of independent and constrained refinement
Δρ_max_, Δρ_min_ (e Å^−3^)	0.26, −0.44
Absolute structure	Flack *x* determined using 1053 quotients [(*I* ^+^)-(*I* ^-^)]/[(*I* ^+^)+(*I* ^-^)] (Parsons *et al.*, 2013 ▸)
Absolute structure parameter	0.10 (17)

Computer programs: *RAPID-AUTO* (Rigaku, 2009 ▸), *CrystalStructure* (Rigaku, 2019 ▸), *olex2.solve* (Bourhis *et al.*, 2015 ▸), *SHELXL2018/3* (Sheldrick, 2015 ▸) and *OLEX2* (Dolomanov *et al.*, 2009 ▸).

## full crystallographic data

### Crystal data

**Table:**

C_7_H_14_O_6_·H_2_O	*Z* = 2
*M**_r_* = 212.20	*F*(000) = 228
Triclinic, *P*1	*D*_x_ = 1.465 Mg m^−^^3^
*a* = 6.7320 (5) Å	Cu *K*α radiation, λ = 1.54187 Å
*b* = 7.7574 (5) Å	Cell parameters from 5608 reflections
*c* = 10.6128 (8) Å	θ = 4.4–68.4°
α = 82.458 (6)°	µ = 1.15 mm^−^^1^
β = 72.596 (5)°	*T* = 296 K
γ = 65.476 (5)°	Block, clear light colourless
*V* = 481.13 (6) Å^3^	0.1 × 0.1 × 0.1 mm

### Data collection

**Table:**

Rigaku R-AXIS RAPID diffractometer	2751 reflections with *I* > 2σ(*I*)
ω scans	*R*_int_ = 0.045
Absorption correction: multi-scan (ABSCOR; Rigaku, 1995)	θ_max_ = 68.2°, θ_min_ = 4.4°
*T*_min_ = 0.698, *T*_max_ = 1.000	*h* = −8→8
5541 measured reflections	*k* = −9→9
2880 independent reflections	*l* = −12→12

### Refinement

**Table:**

Refinement on *F*^2^	Hydrogen site location: mixed
Least-squares matrix: full	H atoms treated by a mixture of independent and constrained refinement
*R*[*F*^2^ > 2σ(*F*^2^)] = 0.048	*w* = 1/[σ^2^(*F*_o_^2^) + (0.0704*P*)^2^ + 0.106*P*] where *P* = (*F*_o_^2^ + 2*F*_c_^2^)/3
*wR*(*F*^2^) = 0.125	(Δ/σ)_max_ < 0.001
*S* = 1.13	Δρ_max_ = 0.26 e Å^−^^3^
2880 reflections	Δρ_min_ = −0.44 e Å^−^^3^
272 parameters	Absolute structure: Flack *x* determined using 1053 quotients [(*I*^+^)-(*I*^-^)]/[(*I*^+^)+(*I*^-^)] (Parsons *et al.*, 2013)
3 restraints	Absolute structure parameter: 0.10 (17)
Primary atom site location: iterative	

### Special details

**Table:**

Geometry. All esds (except the esd in the dihedral angle between two l.s. planes) are estimated using the full covariance matrix. The cell esds are taken into account individually in the estimation of esds in distances, angles and torsion angles; correlations between esds in cell parameters are only used when they are defined by crystal symmetry. An approximate (isotropic) treatment of cell esds is used for estimating esds involving l.s. planes.
Refinement. H atoms were positioned geometrically (C—H = 0.98, 0.97 or 0.96 Å, and O—H = 0.82 Å) and refined using as riding with *U*_iso_(H) = 1.2*U*_eq_(C(H) or C(H,H) groups) or *U*_iso_(H) = 1.5*U*_eq_(C(H,H,H) or O), allowing for free rotation of the OH groups and crystallization water molecules (O3(H3A,H3B) and O4(H4A,H4B)).

### Fractional atomic coordinates and isotropic or equivalent isotropic displacement parameters (Å^2^)

**Table:**

	*x*	*y*	*z*	*U*_iso_*/*U*_eq_	
O3	0.3171 (6)	−0.0810 (5)	0.6471 (5)	0.0640 (11)	
H3A	0.382215	−0.200873	0.640005	0.096*	
H3B	0.176598	−0.058481	0.675652	0.096*	
O4	0.3946 (9)	0.0979 (7)	0.4096 (5)	0.0750 (13)	
H4A	0.330101	0.051068	0.478141	0.113*	
H4B	0.327723	0.101353	0.352282	0.113*	
O11	0.8495 (5)	−0.0155 (4)	0.6286 (3)	0.0423 (7)	
H11	0.842 (12)	0.045 (11)	0.540 (8)	0.08 (2)*	
O12	0.4941 (5)	0.3476 (4)	0.8818 (3)	0.0373 (7)	
O13	0.4864 (4)	0.5035 (4)	0.6457 (3)	0.0361 (6)	
H13	0.483156	0.457826	0.581277	0.054*	
O14	0.8089 (5)	0.6575 (4)	0.6023 (3)	0.0394 (7)	
H14	0.841401	0.738990	0.622566	0.059*	
O15	1.0305 (5)	0.5731 (5)	0.8089 (4)	0.0514 (9)	
H15	1.166424	0.550381	0.782309	0.077*	
O16	0.8842 (5)	0.1669 (4)	0.8198 (3)	0.0382 (7)	
C11	0.6562 (7)	0.0918 (6)	0.7280 (5)	0.0398 (10)	
H11A	0.627277	0.008418	0.801180	0.048*	
H11B	0.525403	0.144048	0.692746	0.048*	
C12	0.6836 (6)	0.2508 (5)	0.7784 (4)	0.0287 (8)	
C13	0.7011 (6)	0.4015 (5)	0.6724 (4)	0.0285 (8)	
H13A	0.812148	0.338344	0.591341	0.034*	
C14	0.7743 (6)	0.5397 (5)	0.7129 (4)	0.0279 (8)	
H14A	0.652616	0.617405	0.785360	0.033*	
C15	0.9857 (7)	0.4371 (6)	0.7581 (5)	0.0360 (9)	
H15A	1.113508	0.370500	0.683889	0.043*	
C16	0.9460 (8)	0.2978 (7)	0.8656 (5)	0.0433 (10)	
H16A	1.082904	0.228958	0.894020	0.052*	
H16B	0.826033	0.365798	0.940980	0.052*	
C17	0.4524 (9)	0.2445 (8)	1.0017 (5)	0.0505 (12)	
H17A	0.432290	0.135576	0.983637	0.076*	
H17B	0.317821	0.324424	1.063570	0.076*	
H17C	0.579122	0.204193	1.038288	0.076*	
O21	−0.0310 (5)	1.0841 (4)	0.3619 (3)	0.0416 (7)	
H21	−0.088305	1.198374	0.348118	0.062*	
O22	0.2120 (5)	0.7111 (4)	0.1172 (3)	0.0358 (6)	
O23	0.0693 (5)	0.5868 (5)	0.3561 (3)	0.0382 (7)	
H23	−0.000861	0.594835	0.434404	0.057*	
O24	0.4907 (6)	0.3936 (4)	0.4191 (3)	0.0407 (7)	
H24	0.461466	0.303180	0.412308	0.061*	
O25	0.8573 (5)	0.4521 (5)	0.2383 (4)	0.0505 (9)	
H25	0.950779	0.489488	0.245670	0.076*	
O26	0.3751 (5)	0.8804 (4)	0.1895 (3)	0.0359 (7)	
C21	−0.0209 (7)	0.9850 (6)	0.2564 (5)	0.0399 (10)	
H21A	−0.034544	1.068125	0.179979	0.048*	
H21B	−0.146711	0.945625	0.280896	0.048*	
C22	0.2012 (6)	0.8120 (5)	0.2214 (4)	0.0299 (8)	
C23	0.2295 (6)	0.6684 (5)	0.3348 (4)	0.0275 (8)	
H23A	0.200807	0.734529	0.415127	0.033*	
C24	0.4663 (7)	0.5142 (5)	0.3059 (4)	0.0295 (8)	
H24A	0.490914	0.439397	0.230901	0.035*	
C25	0.6379 (6)	0.5996 (6)	0.2730 (5)	0.0348 (9)	
H25A	0.621075	0.667012	0.349958	0.042*	
C26	0.5985 (7)	0.7374 (6)	0.1591 (5)	0.0424 (10)	
H26A	0.706826	0.796123	0.138250	0.051*	
H26B	0.623581	0.668881	0.081840	0.051*	
C27	0.1985 (9)	0.8085 (7)	−0.0053 (4)	0.0485 (12)	
H27A	0.243717	0.718954	−0.073325	0.073*	
H27B	0.297456	0.874770	−0.027087	0.073*	
H27C	0.045401	0.897626	0.001903	0.073*	

### Atomic displacement parameters (Å^2^)

**Table:**

	*U*^11^	*U*^22^	*U*^33^	*U*^12^	*U*^13^	*U*^23^
O3	0.0457 (19)	0.048 (2)	0.090 (3)	−0.0091 (17)	−0.0201 (19)	−0.004 (2)
O4	0.098 (3)	0.062 (3)	0.098 (3)	−0.054 (3)	−0.047 (3)	0.016 (3)
O11	0.0536 (18)	0.0270 (15)	0.0462 (17)	−0.0171 (13)	−0.0100 (14)	−0.0037 (13)
O12	0.0428 (15)	0.0325 (15)	0.0320 (14)	−0.0159 (12)	−0.0030 (12)	0.0017 (12)
O13	0.0333 (14)	0.0382 (16)	0.0424 (15)	−0.0142 (12)	−0.0193 (12)	0.0026 (12)
O14	0.0534 (18)	0.0294 (15)	0.0389 (15)	−0.0254 (14)	−0.0056 (13)	0.0026 (12)
O15	0.0384 (15)	0.0462 (19)	0.082 (2)	−0.0216 (14)	−0.0207 (16)	−0.0160 (17)
O16	0.0411 (15)	0.0254 (14)	0.0507 (17)	−0.0103 (12)	−0.0219 (13)	0.0033 (12)
C11	0.043 (2)	0.030 (2)	0.050 (2)	−0.0201 (18)	−0.0071 (19)	−0.0055 (19)
C12	0.0268 (17)	0.0219 (17)	0.0372 (19)	−0.0091 (14)	−0.0091 (15)	−0.0007 (15)
C13	0.0302 (18)	0.0288 (19)	0.0299 (17)	−0.0135 (16)	−0.0102 (14)	−0.0007 (15)
C14	0.0286 (17)	0.0256 (18)	0.0302 (18)	−0.0129 (15)	−0.0048 (15)	−0.0033 (15)
C15	0.0282 (17)	0.031 (2)	0.051 (2)	−0.0111 (16)	−0.0104 (17)	−0.0108 (18)
C16	0.049 (2)	0.040 (2)	0.054 (3)	−0.020 (2)	−0.031 (2)	0.003 (2)
C17	0.063 (3)	0.053 (3)	0.039 (2)	−0.031 (2)	−0.009 (2)	0.005 (2)
O21	0.0458 (16)	0.0250 (14)	0.0488 (18)	−0.0019 (13)	−0.0207 (13)	−0.0058 (13)
O22	0.0518 (16)	0.0281 (14)	0.0327 (14)	−0.0150 (13)	−0.0201 (12)	0.0003 (11)
O23	0.0395 (15)	0.0497 (17)	0.0363 (14)	−0.0298 (14)	−0.0083 (12)	0.0002 (13)
O24	0.0595 (18)	0.0286 (15)	0.0457 (16)	−0.0187 (14)	−0.0329 (14)	0.0089 (13)
O25	0.0264 (14)	0.0376 (17)	0.086 (2)	−0.0052 (12)	−0.0183 (15)	−0.0139 (17)
O26	0.0357 (15)	0.0260 (14)	0.0492 (17)	−0.0154 (12)	−0.0142 (13)	0.0061 (12)
C21	0.035 (2)	0.029 (2)	0.052 (3)	−0.0013 (17)	−0.0217 (18)	−0.0054 (18)
C22	0.0272 (18)	0.028 (2)	0.036 (2)	−0.0089 (15)	−0.0130 (16)	−0.0019 (16)
C23	0.0271 (17)	0.0285 (19)	0.0293 (18)	−0.0117 (15)	−0.0100 (14)	−0.0002 (15)
C24	0.0337 (18)	0.0232 (18)	0.0335 (19)	−0.0082 (15)	−0.0157 (15)	−0.0015 (15)
C25	0.0250 (17)	0.025 (2)	0.053 (2)	−0.0048 (15)	−0.0132 (17)	−0.0078 (17)
C26	0.030 (2)	0.040 (2)	0.055 (3)	−0.0174 (18)	−0.0025 (19)	0.001 (2)
C27	0.064 (3)	0.048 (3)	0.033 (2)	−0.018 (2)	−0.022 (2)	0.007 (2)

### Geometric parameters (Å, º)

**Table:**

O3—H3A	0.8500	C17—H17B	0.9600
O3—H3B	0.8500	C17—H17C	0.9600
O4—H4A	0.8502	O21—H21	0.8200
O4—H4B	0.8500	O21—C21	1.409 (5)
O11—H11	1.00 (8)	O22—C22	1.405 (5)
O11—C11	1.417 (5)	O22—C27	1.423 (5)
O12—C12	1.407 (5)	O23—H23	0.8200
O12—C17	1.429 (5)	O23—C23	1.413 (5)
O13—H13	0.8200	O24—H24	0.8200
O13—C13	1.425 (4)	O24—C24	1.429 (5)
O14—H14	0.8200	O25—H25	0.8200
O14—C14	1.414 (4)	O25—C25	1.417 (5)
O15—H15	0.8200	O26—C22	1.413 (5)
O15—C15	1.416 (5)	O26—C26	1.420 (5)
O16—C12	1.411 (5)	C21—H21A	0.9700
O16—C16	1.429 (6)	C21—H21B	0.9700
C11—H11A	0.9700	C21—C22	1.519 (5)
C11—H11B	0.9700	C22—C23	1.528 (5)
C11—C12	1.505 (6)	C23—H23A	0.9800
C12—C13	1.529 (5)	C23—C24	1.512 (5)
C13—H13A	0.9800	C24—H24A	0.9800
C13—C14	1.505 (5)	C24—C25	1.493 (6)
C14—H14A	0.9800	C25—H25A	0.9800
C14—C15	1.503 (5)	C25—C26	1.514 (6)
C15—H15A	0.9800	C26—H26A	0.9700
C15—C16	1.508 (6)	C26—H26B	0.9700
C16—H16A	0.9700	C27—H27A	0.9600
C16—H16B	0.9700	C27—H27B	0.9600
C17—H17A	0.9600	C27—H27C	0.9600
			
H3A—O3—H3B	104.5	H17B—C17—H17C	109.5
H4A—O4—H4B	104.5	C21—O21—H21	109.5
C11—O11—H11	111 (4)	C22—O22—C27	117.3 (3)
C12—O12—C17	117.3 (3)	C23—O23—H23	109.5
C13—O13—H13	109.5	C24—O24—H24	109.5
C14—O14—H14	109.5	C25—O25—H25	109.5
C15—O15—H15	109.5	C22—O26—C26	114.6 (3)
C12—O16—C16	114.6 (3)	O21—C21—H21A	109.5
O11—C11—H11A	109.0	O21—C21—H21B	109.5
O11—C11—H11B	109.0	O21—C21—C22	110.9 (3)
O11—C11—C12	112.8 (3)	H21A—C21—H21B	108.1
H11A—C11—H11B	107.8	C22—C21—H21A	109.5
C12—C11—H11A	109.0	C22—C21—H21B	109.5
C12—C11—H11B	109.0	O22—C22—O26	112.4 (3)
O12—C12—O16	111.9 (3)	O22—C22—C21	111.3 (3)
O12—C12—C11	111.3 (3)	O22—C22—C23	104.6 (3)
O12—C12—C13	105.2 (3)	O26—C22—C21	106.0 (3)
O16—C12—C11	106.1 (3)	O26—C22—C23	110.0 (3)
O16—C12—C13	110.3 (3)	C21—C22—C23	112.7 (3)
C11—C12—C13	112.2 (3)	O23—C23—C22	109.2 (3)
O13—C13—C12	109.5 (3)	O23—C23—H23A	108.8
O13—C13—H13A	108.7	O23—C23—C24	109.6 (3)
O13—C13—C14	108.8 (3)	C22—C23—H23A	108.8
C12—C13—H13A	108.7	C24—C23—C22	111.5 (3)
C14—C13—C12	112.4 (3)	C24—C23—H23A	108.8
C14—C13—H13A	108.7	O24—C24—C23	109.4 (3)
O14—C14—C13	107.0 (3)	O24—C24—H24A	109.3
O14—C14—H14A	109.1	O24—C24—C25	109.4 (3)
O14—C14—C15	111.6 (3)	C23—C24—H24A	109.3
C13—C14—H14A	109.1	C25—C24—C23	110.1 (3)
C15—C14—C13	110.8 (3)	C25—C24—H24A	109.3
C15—C14—H14A	109.1	O25—C25—C24	108.6 (3)
O15—C15—C14	107.9 (3)	O25—C25—H25A	109.6
O15—C15—H15A	110.1	O25—C25—C26	110.9 (3)
O15—C15—C16	109.7 (4)	C24—C25—H25A	109.6
C14—C15—H15A	110.1	C24—C25—C26	108.5 (3)
C14—C15—C16	108.9 (3)	C26—C25—H25A	109.6
C16—C15—H15A	110.1	O26—C26—C25	111.7 (3)
O16—C16—C15	110.9 (4)	O26—C26—H26A	109.3
O16—C16—H16A	109.5	O26—C26—H26B	109.3
O16—C16—H16B	109.5	C25—C26—H26A	109.3
C15—C16—H16A	109.5	C25—C26—H26B	109.3
C15—C16—H16B	109.5	H26A—C26—H26B	108.0
H16A—C16—H16B	108.1	O22—C27—H27A	109.5
O12—C17—H17A	109.5	O22—C27—H27B	109.5
O12—C17—H17B	109.5	O22—C27—H27C	109.5
O12—C17—H17C	109.5	H27A—C27—H27B	109.5
H17A—C17—H17B	109.5	H27A—C27—H27C	109.5
H17A—C17—H17C	109.5	H27B—C27—H27C	109.5
			
O11—C11—C12—O12	−176.7 (3)	O21—C21—C22—O22	179.4 (3)
O11—C11—C12—O16	−54.8 (4)	O21—C21—C22—O26	−58.1 (4)
O11—C11—C12—C13	65.6 (4)	O21—C21—C22—C23	62.3 (5)
O12—C12—C13—O13	−50.8 (4)	O22—C22—C23—O23	−52.9 (4)
O12—C12—C13—C14	70.2 (4)	O22—C22—C23—C24	68.4 (4)
O13—C13—C14—O14	−65.1 (3)	O23—C23—C24—O24	−64.1 (4)
O13—C13—C14—C15	173.1 (3)	O23—C23—C24—C25	175.6 (3)
O14—C14—C15—O15	67.4 (4)	O24—C24—C25—O25	63.4 (4)
O14—C14—C15—C16	−173.6 (3)	O24—C24—C25—C26	−176.0 (3)
O15—C15—C16—O16	175.8 (3)	O25—C25—C26—O26	176.8 (3)
O16—C12—C13—O13	−171.6 (3)	O26—C22—C23—O23	−173.8 (3)
O16—C12—C13—C14	−50.6 (4)	O26—C22—C23—C24	−52.5 (4)
C11—C12—C13—O13	70.4 (4)	C21—C22—C23—O23	68.1 (4)
C11—C12—C13—C14	−168.6 (3)	C21—C22—C23—C24	−170.6 (3)
C12—O16—C16—C15	−60.5 (5)	C22—O26—C26—C25	−59.1 (5)
C12—C13—C14—O14	173.5 (3)	C22—C23—C24—O24	174.8 (3)
C12—C13—C14—C15	51.7 (4)	C22—C23—C24—C25	54.6 (4)
C13—C14—C15—O15	−173.4 (3)	C23—C24—C25—O25	−176.3 (3)
C13—C14—C15—C16	−54.4 (4)	C23—C24—C25—C26	−55.8 (4)
C14—C15—C16—O16	57.9 (5)	C24—C25—C26—O26	57.6 (5)
C16—O16—C12—O12	−61.3 (4)	C26—O26—C22—O22	−60.8 (4)
C16—O16—C12—C11	177.1 (4)	C26—O26—C22—C21	177.4 (4)
C16—O16—C12—C13	55.4 (4)	C26—O26—C22—C23	55.3 (4)
C17—O12—C12—O16	−53.3 (5)	C27—O22—C22—O26	−58.5 (4)
C17—O12—C12—C11	65.2 (5)	C27—O22—C22—C21	60.3 (5)
C17—O12—C12—C13	−173.1 (4)	C27—O22—C22—C23	−177.8 (3)

### Hydrogen-bond geometry (Å, º)

**Table:**

*D*—H···*A*	*D*—H	H···*A*	*D*···*A*	*D*—H···*A*
O4—H4*A*···O3	0.85	1.94	2.729 (7)	154
O11—H11···O21^i^	1.00 (8)	1.87 (8)	2.799 (4)	154 (6)
O13—H13···O24	0.82	1.83	2.643 (4)	169
O14—H14···O11^ii^	0.82	1.94	2.719 (4)	159
O15—H15···O13^iii^	0.82	2.12	2.898 (4)	158
O21—H21···O25^iv^	0.82	2.10	2.874 (4)	157
O23—H23···O14^v^	0.82	1.84	2.653 (4)	169
O24—H24···O4	0.82	1.83	2.650 (5)	176
O25—H25···O23^iii^	0.82	1.97	2.709 (5)	150

Symmetry codes: (i) *x*+1, *y*−1, *z*; (ii) *x*, *y*+1, *z*; (iii) *x*+1, *y*, *z*; (iv) *x*−1, *y*+1, *z*; (v) *x*−1, *y*, *z*.
